# Historical and projected datasets of the United States electricity-water-climate nexus

**DOI:** 10.1016/j.dib.2021.107399

**Published:** 2021-09-20

**Authors:** Julian Fulton, Ying Jin

**Affiliations:** a Department of Environmental Studies, California State University Sacramento, USA; b Department of Computer Science, California State University Sacramento, USA

**Keywords:** Energy-water nexus, Electricity generation, Greenhouse gas emissions, Power system water consumption and withdrawal

## Abstract

This article describes datasets that were produced in connection with the research article: “Visualizing the United States electricity-water-climate nexus” published in *Environmental Modeling and Software* (https://doi.org/10.1016/j.envsoft.2021.105128). Data cover 9,961 individual power plants across the United States, including monthly values for electricity generation, greenhouse gas emissions, water withdrawal, and water consumption between 2003 and 2020, as well as projections out to 2050. Data were retrieved from publicly available sources and processed for the purpose of providing plant-level information that can be aggregated according to various user needs. Power plant information was retrieved from the US EPA Facility Registry Service (FRS) web service through the filter of “EIA860.” For these plants, we retrieved electricity generation, greenhouse emission, water consumption, and water withdrawal of each plant from heterogeneous data sources, including web services and files, clean and process them, and save them in our database tables. We filled remaining data gaps using a coefficient-based approach. This data article describes metadata and methods for producing the historical and projected datasets in the format of CSV files. The datasets are beneficial for researchers to view electricity generation in the context of emissions and water usage at the granularity of power plants, such as for data analysis and machine learning. These data also can be aggregated to different spatial scales, such as watershed, county, state, and national level, according to different analytical needs. In addition, decision makers can use these data for future energy and resource allocations with the awareness of emission and water constraints.

## Specifications Table

**Table:** 

Subject	Renewable Energy, Sustainability and the Environment
Specific subject area	Energy-Water Nexus
Type of data	CSV files
How data were acquired	Raw data were retrieved from heterogeneous, publicly available data sources, including web services and files.
Data format	Mix of raw and processed data
Parameters for data collection	We prioritized publicly available data from government sources that were accessible with web services through computer programming interfaces. When web services were not available, we used downloadable data. To fill data gaps, we also used water use coefficients from existing peer-reviewed literature, as discussed in the methods section.
Description of data collection	We collected the following information through web services: Facility Information: EPA Facility Registry Service (FRS) web service [1] Historical electricity generation data: U.S. Energy Information Administration (EIA) Plant-level Generation web service [2] Historical emission data: EPA GHGRP (EnviroFacts) greenhouse emissions web service [3] Projected electricity generation data: EIA Annual Energy Outlook 2019 [4] We collected the following through CSV files: Historical water consumption and withdrawal data: EIA thermoelectric cooling water data [5]
Data source location	Primary data sources: • US Environmental Protection Agency, “Facility Registry Service,” from: https://www.epa.gov/frs/frs-data-resources [1] • Energy Information Administration (EIA), “Open data”, from: https://www.eia.gov/opendata/qb.php?category=1017 [2] • US Environmental Protection Agency “Greenhouse Gas Reporting Program (GHGRP),” from: https://www.epa.gov/ghgreporting/ghgreporting-program-data-sets [3] • Energy Information Administration (EIA), “Annual Energy Outlook 2019,” from: https://www.eia.gov/outlooks/archive/aeo19/ [4] • Energy Information Administration (EIA), “Thermoelectric cooling water data”, from: https://www.eia.gov/electricity/data/water/ [5]
Data accessibility	Repository name: HydroShare Direct URL to data: https://www.hydroshare.org/resource/3fd8fd7df98448a097d49921b0f9712c/ Instructions for accessing these data: Go to the “content” section to download the CSV files.
Related research article	J. Fulton, Y. Jin, Visualizing the United States electricity-water-climate nexus, Environ. Model. Softw. 143 (2021) 105128. https://doi.org/10.1016/j.envsoft.2021.105128.

## Value of the Data

• Water use in the electricity sector is not fully captured in existing energy-water assessments, which can be complemented by our modeling approach and data provided in HydroShare. Our data sets cover 9961 power plants with different plant types across the United States, from 2003 to 2050. It has the granularity of monthly data at the plant level that can be aggregated to different levels to satisfy various user requirements.
• Potential Users of these data include:
  ○ Power systems researchers seeking to extend or verify their models in terms of water usage.
  ○ Government agencies tasked with monitoring current power systems and planning for environmental impacts of future ones.
  ○ Water resource stakeholders tasked with inventorying power system water use at various geographic scales.
  ○ Electric utilities seeking to inventory water demand from power sources and assess portfolio options in terms of water use and greenhouse gas emissions.
  ○ Electricity Balancing Authorities and Regional Entities tasked with ensuring reliability of and managing natural resource constraints on power systems.
• Potential data uses:
  ○ Users can use the data directly for plant-level generation, emission, water consumption, and water withdrawal, for data analysis and machine learning.
  ○ Users can aggregate the data into different spatial scales including watershed (HUC-8), county, state, and national.
  ○ Once users aggregate the data into the HUC level, users can compare water consumption and water withdrawal with water availability and other hydrologic metrics from other models.

## 1 Data Description

(1) CSV file named historical.csv:
  This file contains the historical data from 2003 to 2020. Table 1 shows the description of each field.

**Table:** 

Field	Description
plantCode	unique identifier of each power plant (same as EIA plant code)
primaryName	power plant name
naicsCode	NAICS code of the plant
registryId	registry identifier in the EPA FRS system
fipsCode	5-digit FIPS code (state and county) where the plant is located
facAddr	street address of the power plant
cityName	city of the power plant
stateName	state where the power plant is located
postalCode	postal code of the power plant
latitude	latitude where the power plant is located
longitude	longitude where the power plant is located
GEOID	5-digit GEOID (state and county) where the power plant is located
CountyState1	county and state where the power plant is located
CountyState2	county and state abbreviation where the power plant is located
HUC8Code	HUC8 Code where the power plant is located
HUC8Name	HUC8 Name where the power plant is located
HUC8Acres	acres of the HUC8 area
genYear	generation year
genMonth	generation month
plantType	dominant power plant type (fuel-prime mover)
fuelType	dominant fuel type of the power plant
coolingSystemType	dominant cooling system type, e.g. open- or closed-loop
waterType	dominant cooling water type, e.g. fresh, saline, or brackish
waterSource	dominant cooling water source, e.g. ground, surface, or reclaimed
waterSourceName	name of the cooling water source
generation	electricity generation in the given year and month (MWh)
emissions	greenhouse gas emission in the given year and month (MtCO2e)
emissionsDerived	emissions flag: 0=data that is based on yearly raw data; 1=derived data produced by coefficient
waterWithdrawal	water withdrawal in the given year and month (MGal)
waterWithdrawalDerived	water withdrawal flag: 0=raw data; 1=derived data produced by dynamic coefficient; 2=derived data produced by static coefficient except where fuel type is “water”; 3=derived data produced by static coefficient where fuel type is “water”
waterConsumption	water consumption in the given year and month (MGal)
waterConsumptionDerived	water consumption flag: 0=raw data; 1=derived data produced by dynamic coefficient; 2=derived data produced by static coefficient except where fuel type is “water”; 3=derived data produced by static coefficient where fuel type is “water”
Hydropowercentroid	georeferenced centroid for hydroelectric power plants

(2) CSV file named projected.csv:
  This file covers projected data from 2020 to 2050.
  The following fields have the same explanations as the historical data (see above). plantCode, primaryName, naicsCode, registryId, facAddr cityName, stateName, postalCode, latitude, longitude,GEOID,CountyState1,CountyState2, HUC8Code, HUC8Name, HUC8Acres, genYear, genMonth, FuelMover, plantType, fuelType, coolingSystemType, waterType, waterSource, waterSourceName, generation, emissions, waterWithdrawal, waterWithdrawalDerived,waterConsumption, waterConsumptionDerived.

**Table 1:** Static coefficients of water withdrawal and consumption by plant type, adapted from a study by Grubert and Sanders [6]. Lifecycle stages refer water uses that take place at the power plant water related to the pre-conversion (1), conversion (2), and post-conversion (3) stages of electricity production, as specified in the study.

	Static_Consumption_Ratio	Static_Withdrawal_Ratio	Lifecycle
Plant Type	(Gal/MWh)	(Gal/MWh)	stages
AB-ST	78	3,156	2,3
BFG-OT	245	11,054	2,3
BFG-ST	245	11,054	2,3
BIT-GT	245	11,054	2,3
BIT-ST	245	11,054	2,3
BLQ-ST	78	3,156	2,3
DFO-CA	13	90	2
DFO-CT	13	90	2
DFO-GT	13	90	2
DFO-HY	13	90	2
DFO-IC	13	90	2
DFO-ST	13	90	2
GEO-BT	2,950	2,953	1,2
GEO-ST	2,950	2,953	1,2
JF-GT	13	90	2
JF-IC	13	90	2
KER-GT	13	90	2
LFG-CT	44	1,631	2
LFG-FC	44	1,631	2
LFG-GT	44	1,631	2
LFG-IC	44	1,631	2
LFG-ST	44	1,631	2
LIG-ST	407	20,875	2,3
MSB-ST	78	3,156	2,3
MWH-BA	-	-	
MWH-FW	-	-	
NG-CA	39	744	2
NG-CS	39	744	2
NG-CT	39	744	2
NG-FC	39	744	2
NG-GT	39	744	2
NG-IC	39	744	2
NG-ST	39	744	2
NUC-ST	573	24,642	2,3
OBG-CT	44	1,631	2
OBG-FC	44	1,631	2
OBG-GT	44	1,631	2
OBG-IC	44	1,631	2
OBG-ST	44	1,631	2
OBL-GT	78	3,156	2,3
OBS-ST	78	3,156	2,3
OG-GT	39	744	2
OG-IC	39	744	2
OG-ST	39	744	2
OTH-GT	-	-	
OTH-OT	-	-	
OTH-ST	-	-	
PC-OT	13	90	2
PC-ST	13	90	2
PUR-ST	-	-	
RC-ST	245	11,054	2,3
RFO-CT	13	90	2
RFO-ST	13	90	2
SGC-CA	245	11,054	2,3
SGC-CT	245	11,054	2,3
SGP-CT	13	90	2
SLW-ST	78	3,156	2,3
SUB-ST	436	20,658	2,3
SUN-CP	938	1,566	1,2
SUN-PV	2	2	1
SUN-ST	938	1,566	1,2
TDF-ST	78	3,156	2,3
WAT-HY	See Table 2		1
WAT-PS	See Table 2		1
WC-ST	245	11,054	2,3
WDL-ST	78	3,156	2,3
WDS-OT	78	3,156	2,3
WDS-ST	78	3,156	2,3
WH-OT	-	-	
WH-ST	-	-	
WND-WS	3	30	1
WND-WT	3	30	1
WO-CT	13	90	2
WO-GT	13	90	2
  The only new field is emmCode. EIA AEO 2019 provides yearly generation data until 2050 under different energy “cases” (i.e. scenarios) for each Electric Market Module (EMM) region for each fuel mover. 22 EMM regions are in the AEO 2019, corresponding to the North American Electric Reliability Corporation (NERC) and Independent System Operator (ISO) regions. projected.csv includes the electricity generation projections based on the EIA Reference Case, which includes the most likely prediction based on the current information, compared to other cases.

## 2 Experimental Design, Materials and Methods

We develop different computer programs to retrieve and clean the data from heterogeneous data sources. The data are stored in database tables using Microsoft SQL server. For any facilities reported to EIA FRS by the filter of “EIA 860”, we retrieve the power plants and saved the details of each plant in the Facility table. Only power plants in this table are included in our system and presented in the two CSV files in HydroShare repository. The CSV files were produced by querying the results of joining multiple database tables. This section describes our data processing methods for two components: historical data and projected data.

(1) Historical data (2003-2020)
  This section corresponds to the data in the file of “historical.csv”
  This CSV file only has records when a plant from the Facility table has electricity generation reported in a given year and month (i.e. no generation produces no record). In addition to the generation information, emissions and water usage information in this given year and month is also presented in the same row.

• Greenhouse Gas Emissions
  Emission data that is retrieved from GHG [3] is yearly data. We disaggregate it to monthly data and handle gap years using a coefficient approach. Firstly, we calculate the ratios:

***EmissionCoef_year(i)_  = (Emission _year(i)_/Generation _year(i)_)***
A gap year means that a plant does not have any emission data in a specific year but it mayhave emissions in other years. To estimate the unavailable emission data for the gap year,the emission coefficient value is further processed as follows:
If emYear <= minimal year in the available data
then
***EmissionCoef_unavailableYear_ = EmissionCoef_minYear_***
else
***EmissionCoef_unavailableYear_ = EmissionCoef_maxYear_***
Next, this formula converts yearly emission value to monthly:
Emission_year(i), month(j)_ = EmissionCoef_year(i-1), month(j)_ * Generation_year(i), month(j)_
Database stored procedures are used to produce monthly emission.

• Water Consumption and Withdrawal
  ○ Raw Data
    After retrieving available reported water consumption and withdrawal data from EIA's thermoelectric cooling water CSV files [5], we aggregate the consumption and withdrawal values, respectively, grouping them by plant code, year, and month. Each aggregate result is presented as one row in the file of historical.csv with a derived flag of 0, which signifies that this water consumption and withdrawal are raw data.
  ○ Dynamic Coefficients

For any power plant that has been reported in EIA's thermoelectric cooling water CSV files, but has a gap year, we use the dynamic coefficient approach for the gap year.
Firstly, we use the following database query to calculate the dynamic coefficient as the ratio of known monthly water withdrawal (or consumption) to corresponding monthly generation:
*Select w.plantCode, SUM(w.waterConsumption)/SUM(g.generation) as waterConsumpPerGen, SUM(w.waterWithdrawal)/SUM(g.generation) as waterWithdrawPerGen from cooling_summary_raw_per_year as w, [genPerYear] as g where w.plantCode = g.plantCode and w.usageYear = g.genYear and g.generation !=0 Group by w.plantCode*
Next, we use database stored procedures to multiple this coefficient by the known monthly generation value to produce water consumption and withdrawal values:
WaterConsumption_year(i),month(j)_ = WaterConsumption_ratio * Generation_year(i),month(j)_
WaterWithdrawal_year(i),month(j)_ = WaterWithdrawal_ratio * Generation_year(i),month(j)_
In this case, the value of “waterWithdrawalDerived” field (i.e. flag) is “1”. Similarly, “waterConsumptionDerived” field is also “1”.

  ○ Static Coefficients

Only about 10% of power plants have raw water use data (those that report to EIA are larger (> 100MW) fossil, nuclear, biomass, and solar thermal plants); water use at the remaining 90% is estimated using static coefficients from a comprehensive study of water use in the U.S. energy sector [6] as well as another study specifically for hydroelectric facilities [7].
First, since many power plants have multiple fuel and prime mover types, we characterize plant type (denoted as *plantType* in the historic.csv file) by querying yearly generation for each fuel-prime mover combination at each plant and choosing the plant type with the largest generation value. Next (except for hydroelectric power plants), each power plant's monthly generation is multiplied by the corresponding coefficient – notated as Static_Consumption_Ratio and Static_Withdrawal_Ratio in Table 1 – using the following stored procedures.
WaterConsumption_year(i),month(j)_ = Static_Consumption_Ratio *Generation_year(i),month(j)_/1,000,000
WaterWithdrawal_year(i),month(j)_ = Static_Withdrawal_Ratio * Generation_year(i),month(j)_/1,000,000

  ○ Static Hydropower Coefficients

Hydroelectric power plants (i.e. those with a fuel type of “water”) were assigned regional withdrawal and consumption coefficients from hydropower-specific study by Grubert [7], which account for evaporation and seepage of reservoirs, as shown in Table 2.

**Table 2:** Regional (centroid-based) static withdrawal and consumption coefficients for hydroelectric power plants, adapted from a study by Grubert [7].

	Static_Withdrawal_Ratio and
	Static_Consumption_Ratio
Centroid	(Gal/MWh)
1	19,686
2	31,360
3	4,803
4	1,878
5	6,657
6	285
7	6,800
8	71
9	832
10	499
11	1,308
12	2,140
13	6,633
14	713
15	642
16	24,322
17	21,921
18	(12,625)
19	1,022
20	5,896
21	2,518
22	2,518

• Water type

We retrieve cooling system type, water type, water source, and water source name from the same EIA's thermoelectric cooling water CSV files [5] as water consumption and withdrawal. If a facility has more than one cooling system type, the cooling system type with the maximum total generation is used as the cooling system type, which is described as the dominant cooling system type in the second column in Table 1. Water type, water source, and water source name are produced in the same way. For a gap year, we use either minimal or maximum year of available data to fill in the blank, which has the same logic as processing gap years in emissions.

(2) Projected data (2020 to 2050)
  This section corresponds to the data in the file of projected.csv
  EIA AEO 2019 yearly projections of generation data per EMM region and fuel type. We disaggregated the yearly regional data into plant-level monthly generation data by the following two steps: (1) calculating the percent contribution to the total EMM-regional generation per fuel-type by a power plant for each month in the historic data for year 2018, (2) Apply this percentage to regional data for each future year and month.
  With individual plant-level generation data, we calculate projected emissions similar to the calculation of historical emissions. Specifically, the plant-level coefficient from the most recent year (denoted as “maxYear” below) of emissions data is multiplied by generation of a given month and year to produce the projected corresponding emission value. For example, for a given power plant, if no emission data after 2018 is available, then “max year” of this plant is 2018.

***EmissionCoef_projected_  =*** ***Emission _maxYear_/Generation _maxYear_***

A predicted emission value is the multiplication of the ratio and the predicted generationvalue.

Emission_year(i), month(j)_ = EmissionCoef_projected_ * Generation_year(i), month(j)_

Regarding water consumption and withdrawal, all coefficients are the same as the historicalsystem since it already considering all the available years until 2018. A projected waterconsumption or withdrawal value is the product of the coefficient and the predicted generation value.

The projected cooling system type, water type, water source, and water source name are the same as that in the historical data from the most recent available year for each power plant.

## Ethics Statement

None.

## CRediT authorship contribution statement

**Julian Fulton:** Conceptualization, Methodology, Supervision. **Ying Jin:** Methodology, Software, Data curation.

## Declaration of Competing Interest

This work was supported in part by the United States Environmental Protection Agency Exchange Network Grant Program [Grant number OS-83923301].

The authors declare that they have no known competing financial interests or personal relationships which have or could be perceived to have influenced the work reported in this article.
